# Evidence for a maintenance cost for birds maintaining highly flexible basal, but not summit, metabolic rates

**DOI:** 10.1038/s41598-023-36218-w

**Published:** 2023-06-02

**Authors:** David L. Swanson, Maria Stager, François Vézina, Jin-Song Liu, Andrew E. McKechnie, Reza Goljani Amirkhiz

**Affiliations:** 1 Department of Biology, University of South Dakota, Vermillion, SD USA; 2 Department of Biology, University of Massachusetts, Amherst, MA USA; 3 Département de Biologie, Chimie et Géographie, Université du Québec à Rimouski, Rimouski, QC Canada; 4 School of Life and Environmental Sciences, Wenzhou University, Wenzhou, China; 5 DST‑NRF Centre of Excellence at the Percy FitzPatrick Institute, Department of Zoology and Entomology, University of Pretoria, Private Bag X20, Hatfield, South Africa; 6 South African Research Chair in Conservation Physiology, South African National Biodiversity Institute, P.O. Box 754, Pretoria, 0001 South Africa

**Keywords:** Physiology, Ecology

## Abstract

Reversible phenotypic flexibility allows organisms to better match phenotypes to prevailing environmental conditions and may produce fitness benefits. Costs and constraints of phenotypic flexibility may limit the capacity for flexible responses but are not well understood nor documented. Costs could include expenses associated with maintaining the flexible system or with generating the flexible response. One potential cost of maintaining a flexible system is an energetic cost reflected in the basal metabolic rate (BMR), with elevated BMR in individuals with more flexible metabolic responses. We accessed data from thermal acclimation studies of birds where BMR and/or M_sum_ (maximum cold-induced metabolic rate) were measured before and after acclimation, as a measure of metabolic flexibility, to test the hypothesis that flexibility in BMR (ΔBMR), M_sum_ (ΔM_sum_), or metabolic scope (M_sum_ − BMR; ΔScope) is positively correlated with BMR. When temperature treatments lasted at least three weeks, three of six species showed significant positive correlations between ΔBMR and BMR, one species showed a significant negative correlation, and two species showed no significant correlation. ΔM_sum_ and BMR were not significantly correlated for any species and ΔScope and BMR were significantly positively correlated for only one species. These data suggest that support costs exist for maintaining high BMR flexibility for some bird species, but high flexibility in M_sum_ or metabolic scope does not generally incur elevated maintenance costs.

## Introduction

Optimal allocation of energy is a central component of life history, and adaptive adjustment of metabolic rates to variable energy demands can prominently influence survival and fitness^1^. Phenotypically plastic physiological responses, including organismal metabolic rates, to changing environmental or ecological demands are widespread among living organisms^2,3^. Moreover, individual variation in the capacity for plastic physiological responses may also occur among organisms, suggesting the potential for selection on plasticity^2,4^. Animal metabolic rates are plastic traits and vary predictably in response to variation in energy demands resulting from changing environmental or ecological conditions^5,6^. Reversible variation in physiological traits of adult organisms with environmental or ecological variation is termed phenotypic flexibility^7^. Such flexibility in physiological traits, including metabolic rates, may have positive fitness consequences^8–11^, but may also incur costs that trade-off with the capacity for flexibility. The costs of such flexibility (and phenotypic plasticity generally), however, are poorly known and are recommended targets of future research^2,4^. Dewitt et al.^12^ and Auld et al.^13^ defined five categories of potential costs of phenotypic plasticity. The costs most pertinent to phenotypic flexibility include costs associated with maintaining systems capable of flexible responses as well as sensing fluctuations in the environment and costs associated with producing a flexible response to the fluctuating environment, if such costs exceed those for the production of a fixed trait value^12,13^. One potential cost of maintaining a system capable of flexible metabolic responses could be an energetic cost that is reflected in the basal metabolic rate (BMR), with elevated BMR in individuals with more flexible metabolic responses to environmental variation.

Seasonal metabolic flexibility in ectothermic vertebrates typically involves physiological adjustments that partially or completely compensate for temperature effects on metabolic rates, so acclimation maintains metabolic function across a range of environmental temperatures^14^. Ectothermic vertebrates in cold winter climates also typically downregulate metabolic rates and become dormant or adopt energy conserving strategies during winter^15,16^. In contrast, winter-active endotherms, especially birds from cold winter climates, increase metabolic capacity in response to increases in energy demands for winter thermoregulation^17^. Consequently, climatic variation increases energy demands and elevates, rather than maintains, metabolic capacities in cold winter climates. Thus, birds show a fundamentally different pattern of metabolic flexibility in response to variable climates than vertebrates that downregulate metabolic rates in winter to reduce activity or become dormant over time scales of weeks to months (ectotherms and hibernating mammals). Patterns, mechanisms, and costs of metabolic flexibility in birds are, therefore, also likely to differ markedly from those in vertebrates that adopt energy conservation strategies in winter^18,19^.

Summit metabolic rate (M_sum_ = maximum thermogenic metabolic rate) is positively associated with cold tolerance capacity in birds^20^. In addition, flexibility of metabolic traits (M_sum_ and BMR) varies among individuals and appears to be repeatable in endotherms^21,22^ and so may be subject to selection. Moreover, winter metabolic traits are associated with overwinter survival in birds. Upregulation of winter M_sum_ above threshold values is positively associated with overwinter survival in birds^10,11^. Winter BMR is also associated with overwinter survival in birds, but either in a fluctuating manner, with high BMR favored in cold winters and low BMR favored in mild winters^9^, or showing a pattern of stabilizing or disruptive selection, rather than directional selection^8,23^. In addition, metabolic scope (M_sum _− BMR) defines the capacity of birds to elevate metabolic rate for thermoregulatory purposes, and may be important to cold tolerance capacities^19^. Thus, flexible metabolic responses to temperature variation and cold tolerance capacity are linked to fitness consequences in birds and endotherms in general.

**Figure 1:**
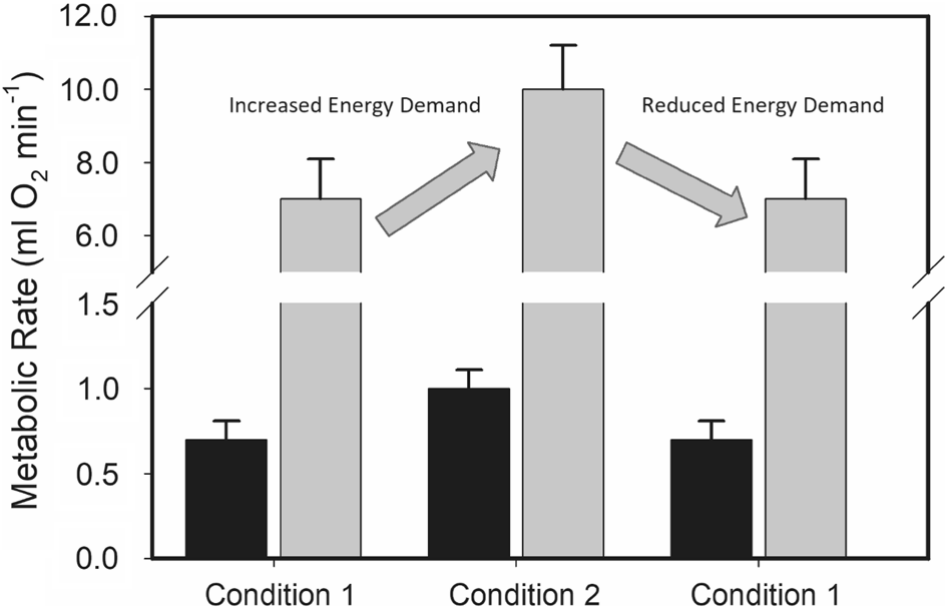
Examples of metabolic flexibility for basal (black) and summit (M_sum_; gray) metabolic rates in endotherms. Condition 1 represents a period of relatively low energy demand (e.g., warm temperatures, non-migratory, non-breeding) and Condition 2 represents a period of elevated energy demands (e.g., cold temperatures, migration, chick rearing). Differing energy demands result in reversible changes of metabolic rates (e.g., typically an upregulation under conditions of increased energy demands). Metabolic flexibility allows moving back and forth between high-energy and low-energy phenotypes as dictated by environmental or ecological demands.

Although definitions of metabolic flexibility may vary, here we define metabolic flexibility as the difference in metabolic rate between two conditions (e.g., winter and summer) differing in energy demands (Fig. 1). A prominent example of metabolic flexibility is the seasonal phenotypes of small resident birds from highly seasonal climates^17^, which typically show winter increases in BMR and M_sum_ to meet thermoregulatory demands of the cold winter climate. Seasonal variation in these metabolic traits, however, is much more variable in birds from milder winter climates^24^, with winter increases, winter decreases, and no seasonal variation patterns all documented^25–29^. Such patterns of seasonal variation in metabolic traits suggest a role for thermoregulatory costs in determining the patterns and magnitude of seasonal metabolic flexibility. Despite the common occurrence of similar seasonal trends in BMR and M_sum_, such similar seasonal trends are not always evident^26,29^ and recent evidence suggests that these two metabolic traits are not tightly phenotypically linked^10,21,30–32^. Nevertheless, enhanced support costs (e.g., digestive capacity to provide substrates for fuel, circulation of oxygen and substrates to thermogenic muscle) for thermogenic capacity in cold winter climates might be expected to increase BMR in winter relative to summer for small birds^17^, so high M_sum_ flexibility might also be expected to incur a maintenance cost.

In the present study, we collected data on flexibility in BMR, M_sum_, and metabolic scope in birds from previously published acclimation studies where metabolic traits were measured both before and after acclimation so that we could calculate individual flexibility in metabolic traits. We then used a General Linear Model approach to test the hypothesis that individual birds with more flexible responses to acclimation for BMR, M_sum_, and/or metabolic scope had higher pre-acclimation BMR. A positive correlation between flexibility in BMR, M_sum_, or metabolic scope and initial BMR would provide support for the hypothesis and suggest that high metabolic flexibility in birds does result in an energetic cost.

## Materials and methods

### Data collection

We performed a literature search in Google Scholar in May 2022 to identify studies that used cold temperature acclimation protocols to measure metabolic flexibility in BMR and/or M_sum_ and metabolic scope from measurements before and after acclimation. We also included studies that employed warm temperature acclimation treatments if the study also included a cold temperature acclimation protocol. We identified eight studies fitting these requirements (Table 1), all eight of which measured BMR flexibility (although Stager et al.^33^ measured but did not publish BMR data) and six of which also measured flexibility in M_sum_. These studies used temperature acclimation periods ranging from 8 days^31^ to 9 weeks^34^. We used raw data for BMR and M_sum_ from individual birds and calculated metabolic flexibility (ΔBMR, ΔM_sum_ or ΔScope) by subtracting pre-acclimation BMR, M_sum_, or metabolic scope from post-acclimation values for these traits, using the absolute value of the difference (typically an increase with cold acclimation and a decrease with warm acclimation) as a measure of metabolic flexibility. If a study included cold, warm, and control (i.e., a room temperature treatment that was equivalent to pre-acclimation temperature exposure) acclimation treatments, we calculated ΔBMR, ΔM_sum_, or ΔScope only from cold and warm acclimation treatments where a flexible change in metabolic traits might be expected. Studies for which a control group was excluded from analyses included van de Ven et al.^26^, Cui et al.^35^, and Stager et al.^33,34^ (cold acclimation only). The Li et al.^36^ study included a warm temperature treatment (35 °C) that was the same as the captivity acclimation temperature, but the authors did vary photoperiod for both temperature treatment groups from that experienced during captivity acclimation, so we retained the warm treatment group from this study in our analyses.

**Table 1:** Statistics for regression analyses for relationships between flexibility in BMR (ΔBMR), summit metabolism (ΔM_sum_), and metabolic scope (ΔScope) vs. pre-acclimation BMR.

Species	n	ΔBMR *P*	ΔM_sum_ *P*	ΔScope *P*	Accl temps (°C)	Reference
House Sparrow	47	0.008 (+)	0.336	0.350	25/− 10	Swanson et al.^38^
Dark-eyed Junco	49	0.312	0.056	0.003	18/− 8	Stager et al.^34^
Junco spp.	48	0.522	0.398	0.556	23/3	Stager et al.^33^
White-throated Sparrow	28	< 0.001 (+)	0.834	0.321	28/− 8	Barceló et al.^32^
White-throated Sparrow*	20	0.947	0.432	0.482	28/− 5	Dubois et al.^31^
Black-capped Chickadee*	24	0.152	0.584	0.759	28/− 5	Dubois et al.^31^
Snow Bunting*	14	0.250	0.200	0.331	28/− 5	Dubois et al.^31^
Red-billed Leothrix	20	0.189	–		35/15	Cui et al.^35^
Chinese Hwamei	30	0.036 (−)	–		35/15	Li et al.^36^
S. Red Bishop	39	0.046 (+)	0.642	0.157	35/10	van de Ven et al.^26^

Acclimation temperatures (Accl) are the cold- and warm-acclimation treatments used in the cited studies. *P* values are provided for the regressions of flexibility in BMR (ΔBMR), summit metabolism (ΔM_sum_), and metabolic scope (ΔScope) vs. pre-acclimation BMR. Signs provide the direction for significant relationships.

*Acclimated for only 8 days. All other studies used temperature acclimation treatment lasting at least 3 weeks.

### Statistical analyses

We used a General Linear Model approach with a Gaussian distribution (because data were continuous) to test whether metabolic flexibility (ΔBMR, ΔM_sum_, or ΔScope) was associated with pre-acclimation BMR. We ran separate models for ΔBMR, ΔM_sum_, and ΔScope. We verified that residuals for both ΔBMR and ΔM_sum_, and ΔScope models were normally distributed visually through a qq-plot and statistically via the Shapiro–Wilk test. We used single models for ΔBMR, ΔM_sum_, and ΔScope, with ΔBMR, ΔM_sum_, or ΔScope as the response variables and pre-acclimation BMR and temperature acclimation treatment (cold and warm categorical variables if the study included both cold and warm acclimation treatments following a captivity acclimation period at room temperature) as predictor variables, with body mass (M_b_) as a covariate. We also included other experimental treatment groups as predictor variables in GLM analyses of metabolic flexibility, such as diet^32^ or photoperiod^36^ acclimation treatments, if such treatments were included in the study. Finally, we included location or subspecies as predictor variables if > 1 location^26^ or > 1 subspecies^33^ were included in the study.

The duration of captivity prior to acclimation treatments may influence BMR and M_sum_ in birds^26,34^ and durations of captivity acclimation prior to pre-acclimation BMR and M_sum_ measurement varied from 10 to 63 days for studies included in our analyses. To address the issue of whether pre-acclimation captivity period differences might confound analyses of metabolic flexibility in this study, we tested whether variation in pre-acclimation metabolic rates differed with the duration of captivity acclimation periods. We calculated the coefficient of variation (CV) for BMR and M_sum_ after the pre-acclimation captivity period as a measure of variation in metabolic traits. Our hypothesis was that as birds acclimated to captivity, individual metabolic rates should converge toward a similar mean BMR and M_sum_ after captivity acclimation, so that the CV for metabolic rates should be negatively correlated with captivity acclimation period. We conducted linear regressions of CVs for both BMR and M_sum_ against duration of captivity acclimation and found no significant relationships between CV and BMR or M_sum_ (Fig. S1), suggesting that the duration of captivity acclimation likely did not influence the magnitude of variation in BMR or M_sum_ among individuals in the present study. Consequently, we did not include pre-acclimation captivity period in the models. We considered *P* < 0.05 as a statistically significant effect for all predictor variables.

Finally, to address the question of whether potential trade-offs or synergies exist between flexibility in BMR and flexibility in M_sum_ or metabolic scope, we calculated Pearson’s correlation coefficients for these relationships for all species and studies reporting these measures. We considered *P* < 0.05 to represent a statistically significant correlation between variables. All GLM analyses were conducted using package “stats” in R (Version 3.6.1)^37^ and CV and correlation analyses were conducted in SigmaStat, Version 3.5 (Systat Software, Inc., San Jose, CA).

## Results

**Figure 2:**
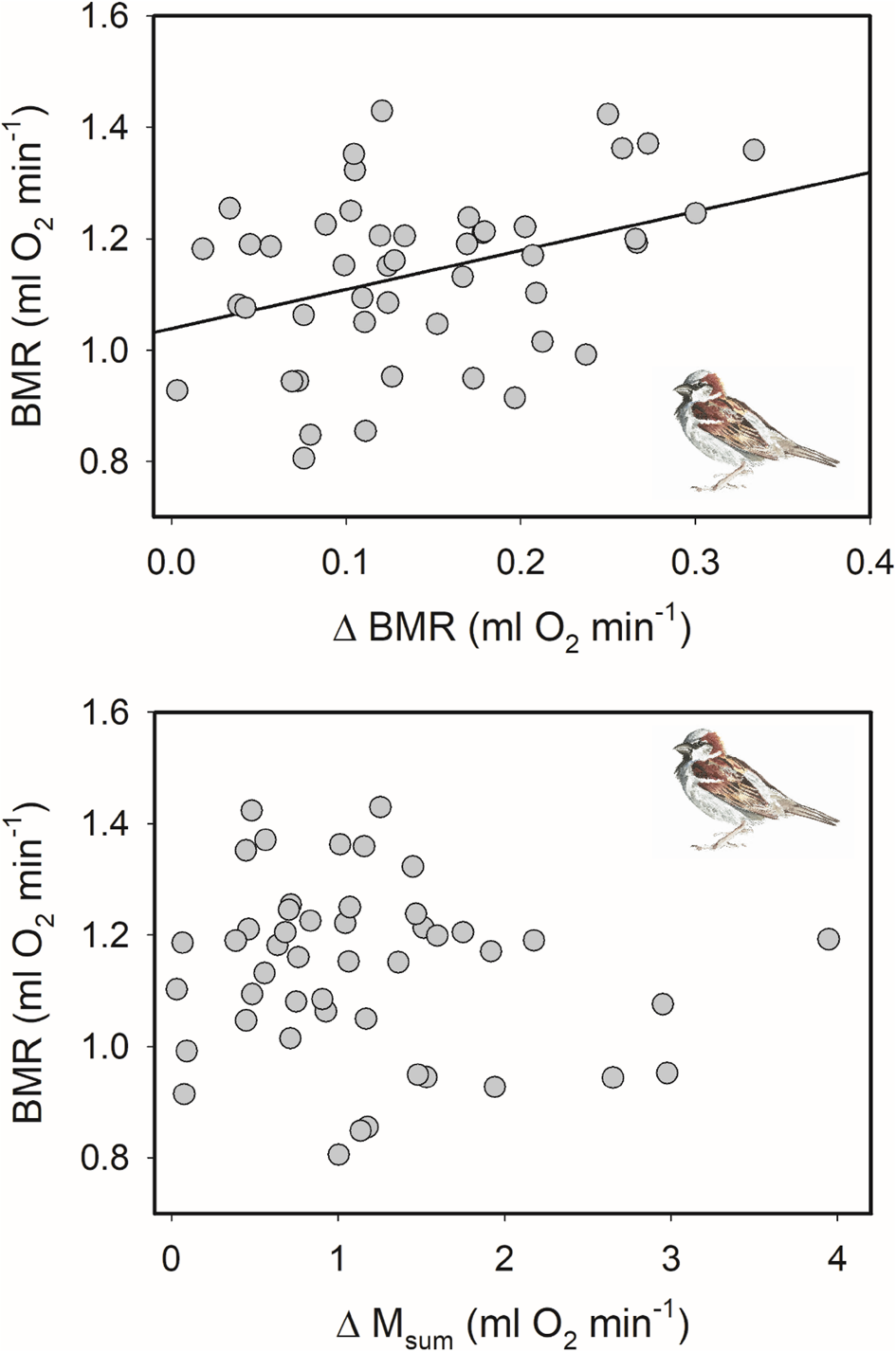
A representative example of relationships between flexibility in BMR (ΔBMR) and summit metabolism (ΔM_sum_) and pre-acclimation BMR in house sparrows (*Passer domesticus*). A significant positive relationship was evident for BMR flexibility but not M_sum_ flexibility. Data from Swanson et al.^38^.

We conducted 10 independent GLMs for ΔBMR using data for eight species (dark-eyed junco, *Junco hyemalis*, and white-throated sparrow, *Zonotrichia albicollis*, occurred in two studies each). For ΔM_sum_ and ΔScope we conducted eight independent GLMs for six species (again dark-eyed junco and white-throated sparrow occurred in two studies each). For the six study species for which acclimation periods lasted at least three weeks, three species showed a positive correlation between BMR and BMR flexibility^26,32,38^, after correcting for body mass, direction of temperature acclimation and other acclimation treatments, indicating that individuals with high initial, pre-acclimation BMR also had greater flexibility in BMR (Table 1, Fig. 2). None of the three species with temperature acclimation treatments lasting for only 8-d showed a significant effect of BMR on BMR flexibility^31^, including white-throated sparrows, which showed a significant positive correlation between ΔBMR and BMR after three weeks of acclimation to similar temperatures^32^. In addition, for the three species acclimated to cold temperatures of 3 °C or lower for 3 weeks or more (white-throated sparrow^32^, dark-eyed junco^33^, and house sparrow, *Passer domesticus*^37^), white-throated and house sparrows showed significant positive correlations between ΔBMR and BMR. In contrast, for the three species acclimated to milder cold treatments of ≥ 10 °C (Southern red bishop, *Euplectes orix*^26^, Chinese hwamei, *Garrulax canorus*^36^, red-billed leiothrix, *Leiothrix lutea*^35^), only the Southern red bishop showed a positive correlation between BMR and ΔBMR, and the Chinese hwamei showed a significant negative correlation between BMR and ΔBMR (Table 1). Thus, a significant positive relationship between ΔBMR and BMR occurred more often with colder acclimation temperatures. We found no significant relationships between BMR and M_sum_ flexibility for any of the study species (Table 1), after correcting for M_b_ and treatment group, although dark-eyed juncos acclimated to − 8 °C showed a trend toward a positive correlation between BMR and ΔM_sum_ (*P* = 0.056)^34^. Similarly, BMR was not significantly correlated with ΔScope, after correcting for other variables, for any study expect for one of the studies with dark-eyed juncos (Table 1), where BMR was significantly positively correlated with ΔScope (*P* = 0.016).

Temperature acclimation treatment (cold vs. warm) was a significant or nearly significant (*P* < 0.06) effector of flexibility in metabolic traits for two of eight tests for BMR flexibility, for three of six tests for M_sum_ flexibility, and for two of six tests for metabolic scope flexibility (Table S1). For the two studies documenting significant associations between temperature acclimation treatment and BMR flexibility, southern red bishops showed greater flexibility for warm acclimated than for cold acclimated birds^26^, but Chinese hwameis showed the reverse trend, irrespective of photoperiod treatment, with greater BMR flexibility in cold acclimated than warm acclimated birds^36^. Southern red bishops^26^, white-throated sparrows^32^, and snow buntings^31^ all showed greater M_sum_ flexibility for warm acclimation (generally downregulation) than for cold acclimation (generally upregulation). For metabolic scope, white-throated sparrows^32^ and snow buntings^31^ showed greater flexibility for warm acclimation than for cold acclimation.

Body mass was a significant predictor of BMR flexibility in three of 10 tests (Table S1), including Chinese hwamei^36^, black-capped chickadee and snow bunting^31^, and was positively correlated with BMR flexibility in all cases. For M_sum_, M_b_ was significant predictor of flexibility for only one of eight tests, being positively correlated with M_sum_ flexibility for the southern red bishop^26^. M_b_ was a significant or nearly significant (*P* ≤ 0.062) effector of ΔScope for three of eight tests, with larger birds showing greater flexibility than smaller birds in all cases (Table S1). Consumption of a high-fiber diet was positively associated with BMR flexibility, but not with flexibility in M_sum_ or metabolic scope, for white-throated sparrows^32^ (Table S1), suggesting that the low digestibility of the high-fiber diet resulted in greater flexibility in BMR. Location or subspecies were not significant effectors of BMR, M_sum_, or metabolic scope flexibility (Table S1) in studies where greater than one location or subspecies were part of the study design^26,33^. BMR flexibility was not significantly correlated with either M_sum_ or metabolic scope flexibility for any species (Table S2).

## Discussion

Flexibility of BMR was positively related to pre-acclimation BMR for three of six species acclimated to temperature treatments for at least 3 weeks, with acclimation to temperatures below 0 °C more likely to produce a positive correlation between BMR flexibility and BMR. These data suggest a maintenance cost of highly flexible BMRs for some, but not all, bird species and, moreover, suggest the cost to BMR flexibility may be especially evident when colder temperatures elicit greater metabolic flexibility. In contrast to BMR flexibility, higher flexibility in M_sum_ or metabolic scope apparently does not generally incur higher maintenance costs, even at colder acclimation temperatures, as only one study of dark-eyed juncos detected a positive correlation between BMR and flexibility in M_sum_ or metabolic scope. This variation in costs of flexibility between BMR and M_sum_ is consistent with previous findings that BMR and M_sum_ are regulated independently rather than being phenotypically linked^21,30–32^. This is, perhaps, not surprising because BMR is thought to be driven primarily by metabolism in central organs (gut, liver, kidney, brain), whereas M_sum_ is driven by peripheral organs (i.e., skeletal muscles) and heart^32,39,40^. In addition, different impacts of seasonal metabolic variation for BMR and M_sum_ on overwinter survival may exist, with directional selection on M_sum_, at least above threshold values^10,11^, but fluctuating, stabilizing or disruptive selection on BMR^8,9,23^. Different selective pressures could result in different seasonal patterns of BMR and M_sum_ variation and different costs for maintaining capacity for flexibility in these two metabolic traits. Finally, similar patterns of flexibility for M_sum_ and metabolic scope, which differ from those for BMR flexibility, are consistent with the results of Stager et al.^19^, who demonstrated that M_sum_, rather than BMR, was the primary determinant of latitudinal patterns of variation of metabolic scope in birds.

The pattern of a positive relationship between maintenance costs (BMR) and flexibility in metabolic rates occurring more often at colder acclimation temperatures (i.e., greater separation between cold and warm acclimation temperatures) is generally consistent with the climatic variability hypothesis (CVH). This hypothesis posits that greater capacities for metabolic flexibility should be favored in highly variable environments^41,42^. Several studies have examined intraspecific associations between geographic variation in flexibility of BMR and environmental heterogeneity in birds, with most regional-scale studies encompassing relatively minor variation in climate among study sites^27,29,42,43^. These studies have provided variable results, with positive^42,44^ or no relationships^27,29,43^ between BMR flexibility and seasonal temperature variation. Similarly, M_sum_ flexibility was not significantly correlated with environmental temperature variability in regional studies of birds across modest seasonal temperature variability^27,29^, but was positively correlated to temperature variability in junco (*Junco hyemalis* and *J. phaeonotus*) populations^33^ across a broader climatic gradient. Thus, while some support for the CVH exists for avian metabolic traits, it does not appear to apply uniformly across locations with different magnitudes of climatic variation. The more frequent positive correlations between flexibility in metabolic traits at colder acclimation temperatures and pre-acclimation BMR in this study suggests that substantial temperature variation may be required to produce sufficient flexibility in BMR to detect a cost for maintaining flexibility in metabolic traits. In other words, the costs of a flexible BMR may be detectable only in studies involving large differences in temperature, an important consideration for future studies testing the CVH and for quantifying the costs of metabolic flexibility.

Positive correlations between BMR flexibility and pre-acclimation BMR were detected only after at least three weeks of acclimation to cold temperatures in this study. Several studies on birds suggest that the full acclimation response may take weeks to develop^34,38,45,46^. Metabolic traits of three species of passerine birds responded to shorter periods (4- and 8-d) of cold acclimation but followed different temporal patterns^31^. However, since the focus of the Dubois et al.^31^ study was the rapidity rather than the magnitude of the acclimation response, it is likely that longer acclimation periods would have yielded different results regarding the magnitude of metabolic flexibility in these species. In addition, thermal history prior to acclimation treatments can influence the rapidity, magnitude, and direction of the flexible metabolic response to temperature^38,47^. Collectively, these data strongly suggest that, although changes in metabolic traits with acclimation can occur within days, development of the full acclimation response often requires periods of weeks. Thus, future studies addressing questions of costs of flexibility should acclimate birds for periods of at least three weeks and likely longer so that full capacities for flexibility to the acclimation treatment may develop.

In addition to BMR, other factors were also associated with metabolic flexibility for at least some birds in the present study. Body mass did not significantly affect BMR, M_sum_, or metabolic scope flexibility in the majority of studies (70% for BMR, 88% for M_sum_ and metabolic scope; Table S1), but when it did, larger birds were capable of greater metabolic flexibility. The mechanisms of this greater metabolic flexibility in larger birds, when present, are unknown at present. Given the trends of decreasing body mass with climate change in birds^48–50^, these results suggest that smaller birds, at least for some species, might be at a disadvantage in dealing with future increases in weather variability forecast by some climate models^51^ and might be more susceptible to phenotypic mismatches with the environment^52^.

For studies documenting significant effects of temperature acclimation on flexibility in M_sum_ or metabolic scope, warm acclimation produced greater flexibility than cold acclimation, suggesting that capacities for downregulating M_sum_ or metabolic scope under warm conditions are greater than for upregulation under cold conditions. Effects of temperature acclimation on BMR were less consistent, with one study supporting greater BMR flexibility with warm acclimation^26^ and one study supporting greater BMR flexibility with cold acclimation^36^. Dubois et al.^31^ documented that BMR changed at similar rates in response to cold and warm acclimation in three passerine species, but that BMR changed more rapidly with temperature acclimation than M_sum_. In addition, BMR was completely reversible when southern red bishops were exposed to cold then warm or vice versa, but M_sum_ was only partly reversible, suggesting slower responses to thermal cues for M_sum_ than for BMR in this species^26^. Swanson et al.^38^ documented metabolic downregulation in winter with both warm and cold temperature acclimation and metabolic upregulation in summer with cold acclimation in house sparrows, suggesting that beginning metabolic rates before acclimation are important determinants of metabolic acclimation responses to temperature. Collectively, these differential responses of BMR and M_sum_ flexibility to warm and cold temperature acclimation further support the idea of independent regulation of BMR and M_sum_ by environmental cues rather than a rigid phenotypic linkage between these two metabolic traits^21,30,32^. Moreover, no significant correlations between flexible responses of BMR and M_sum_ or metabolic scope were detected for any species, suggesting that neither trade-offs nor synergies exist between flexibility in basal and maximal thermogenic metabolic outputs. The absence of correlated flexible responses between basal and maximal thermogenic metabolic rates also supports the conclusion of independent regulation of these metabolic traits.

The mechanisms contributing to a higher BMR in birds with more flexible metabolic phenotypes are unknown, but could involve many factors which, collectively, might allow reduced transition times between new steady state conditions, higher sensitivity to stressors, and more regulatory control sites^53^. These factors potentially include differences in protein turnover rates, membrane permeability, oxidative stress responses, capacities for regulatory gene expression, or adjustment of metabolic enzyme activities. For example, protein turnover rates might be higher for more flexible metabolic phenotypes leading to more rapid upregulation or downregulation of proteins or tissue masses important to adjusting metabolism in response to changing environments. Cellular metabolic rates differ among organs, with central organs (digestive tract, liver, kidney, heart brain) having high cellular metabolic rates than muscles and a strong influence on BMR in endotherms^54^. Associated with higher cellular metabolic rates, protein turnover rates are also higher in central organs than in muscles in birds^55,56^. If protein turnover is more rapid in birds with more flexible metabolic phenotypes, particularly in central organs, this could potentially contribute to elevated BMR for birds with increased BMR flexibility.

The uncoupling of the proton gradient generated by the electron transport system from ATP production in mitochondria, termed membrane “leakiness,” increases with the proportion of polyunsaturated fatty acids in phospholipids of membranes and is positively correlated with metabolic rates in vertebrates^53,57^. Stemming from this relationship, the membrane pacemaker hypothesis suggests that membrane permeability is positively correlated with BMR^57,58^. Mitochondrial proton leak may change with seasonal acclimatization or cold acclimation in birds and is generally higher in tissues in winter relative to summer or in cold-acclimated relative to warm-acclimated birds and is also often positively correlated with BMR^36,59,60^ or M_sum_^61^ in birds. If birds with more flexible phenotypes have leakier membranes, and leakier membranes facilitate more rapid flexible metabolic responses to changes in energy demand, as suggested for mammals by Rolfe and Brown^53^, then this could contribute to a higher BMR in birds with more flexible phenotypes. The mechanisms by which leakier membranes might facilitate metabolic flexibility are not currently known but could potentially involve either adjusting membrane proton flux under conditions of changing energy demands or requiring greater metabolic changes to overcome the higher proton leak.

High or flexible metabolic rates may be associated with high rates of reactive oxygen species production and oxidative stress, although the relationship between oxidative damage and elevated metabolic rates is not consistent among birds or energetically demanding conditions^52,62,63^. Nevertheless, if ROS production increases with the production of a flexible metabolic response, then oxidative damage might occur, unless antioxidant capacity is simultaneously increased. Antioxidant capacity is often increased under conditions of elevated energy demand in birds^52,63^ but acclimatory responses have been little studied in birds generally. Acclimation-induced mismatches between ROS production and antioxidant capacity resulting in oxidative damage might constrain the capacity for acclimation or represent a cost of being mismatched with the environment until a new phenotype can be produced^64,65^. Elevated biosynthetic costs for production of antioxidant enzymes and repair mechanisms in response to metabolic flexibility, as well as costs for maintaining the capacity to rapidly upregulate antioxidant production under changing environmental or ecological conditions, could potentially contribute to a higher BMR in birds with more flexible phenotypes.

If individual, repeatable, differences in gene expression capacity^66–68^ exist in birds, then greater capacity for regulatory gene expression would be expected for individuals with more flexible phenotypes and might also carry an energetic cost that could contribute to BMR. Although some recent avian studies have assessed gene-by-environment interactions by comparing gene expression differences across acclimation treatments where metabolic traits also differ^69^, study of how among-individual differences in gene expression relate to among-individual differences in metabolic flexibility is essentially absent for endotherms^68^. Adaptive individual differences in gene expression may occur in ectotherms in response to environmental stressors^70^, suggesting that among-individual differences in capacities for regulatory gene expression might also be considered a cost of physiological flexibility. If among-individual differences occur in the capacity for regulating gene expression, and if this capacity carries a bioenergetic cost (e.g., maintaining higher levels of pathway intermediates to allow rapid changes in gene expression), such differences could potentially contribute to individual differences in BMR, with higher costs for more flexible phenotypes. In addition, maintaining greater capacities for rapid activation of metabolic enzymes (i.e., more responsive signaling pathways or higher concentrations of precursor zymogens in an inactive state) could also potentially contribute to higher BMR in birds with more flexible phenotypes.

In summary, physiological factors such as organ-specific differences in protein turnover rates, membrane permeability, oxidative stress responses, and capacities for regulation of gene expression or metabolic enzyme activities, show flexibility and individual variation in birds. Consequently, these factors have the potential to contribute to the positive relationship between BMR and BMR flexibility documented for several bird species acclimated to cold or warm conditions in the present study. However, direct demonstration of effects of variation in these factors on BMR or BMR flexibility is lacking. As such, examination of within-individual variation in these factors, which might determine costs to flexibility, how these factors might contribute to BMR, and how they vary with flexible metabolic phenotypes, would be a profitable avenue for further study of constraints on flexible metabolic responses to changing environmental or ecological demands in birds and other organisms.

## Supplementary Information

Supplementary Information.

## Data Availability

Data are available upon request from the corresponding author, David L. Swanson, and are also publicly available in Dryad (https://doi.org/10.5061/dryad.hqbzkh1n2).
