# Supplementary material for: Evidence for a maintenance cost for birds maintaining highly flexible basal, but not summit, metabolic rates
Source: Sci Rep. 2023 Jun 2;13:8968. doi: 10.1038/s41598-023-36218-w (PMC10238479; doi:10.1038/s41598-023-36218-w)
Supplement: Supplementary file 1 — Supplementary Information. [file 41598_2023_36218_MOESM1_ESM.docx]

**Figure S1**. Coefficient of variation in pre-acclimation BMR and M_sum_ vs. the duration of the captivity acclimation period for the bird species in the studies in Table 1. No significant relationships were evident. Regression statistics were – BMR: *F_1,8_* = 0.915, *P* = 0.367; M_sum_: BMR: *F_1,6_* = 2.311, *P* = 0.179.


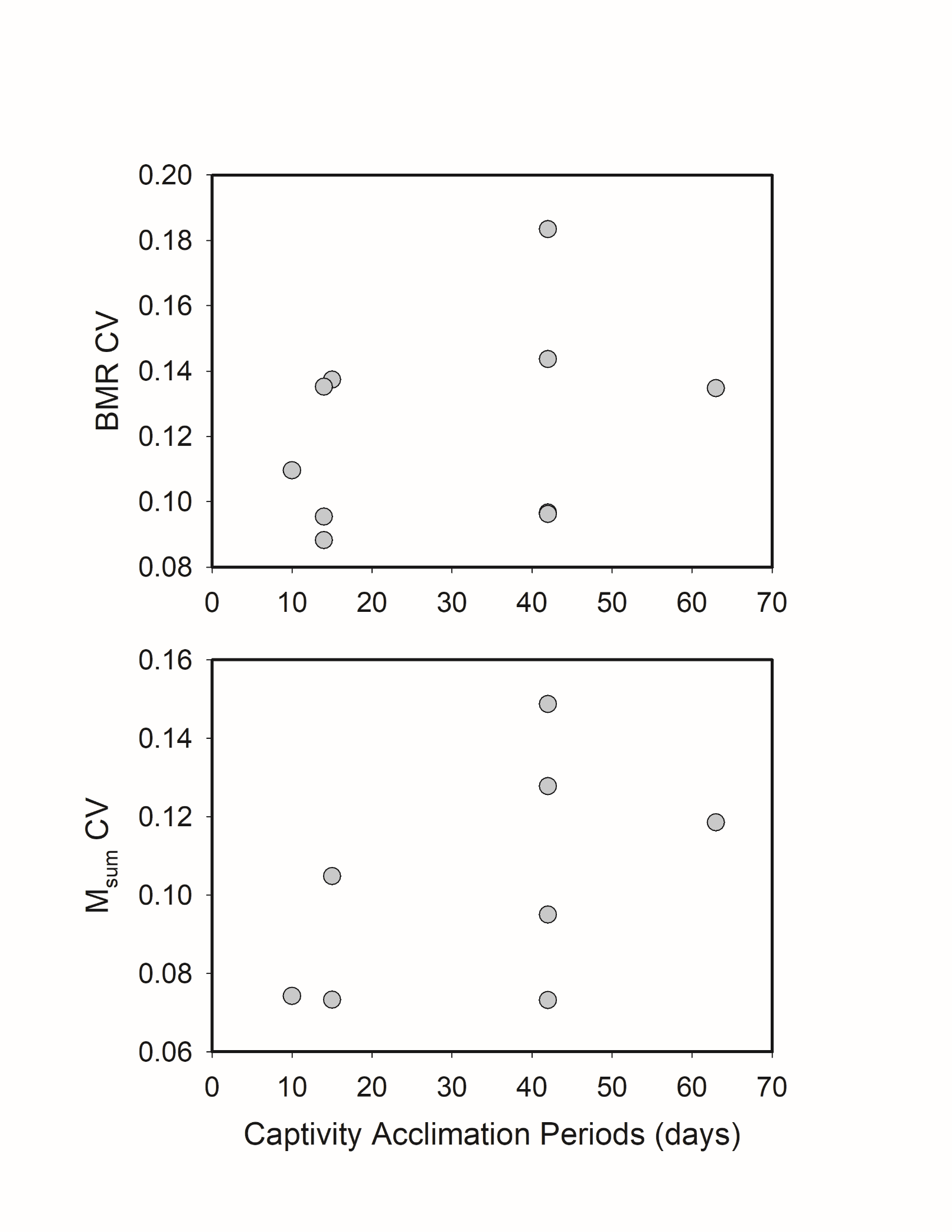


**Table S1**. Results from GLM models with flexibility in metabolic traits (ΔBMR and ΔM_sum_, or ΔScope) as the dependent variable and predictor variables including BMR, body mass (M_b_), temperature acclimation treatment group (TempGrp), and other treatment groups (e.g., photoperiod, diet, location, subspecies).

| **Species** | **n** | **Dependent Variable** | **Predictor Variable** | ***t*** | ***P*** | **Reference** |
| --- | --- | --- | --- | --- | --- | --- |
| Chinese Hwamei | 30 | ΔBMR | M_b_ | 2.209 | 0.037 | Li et al. 2022 |
| *Garrulax canorus* |  |  | BMR | -2.221 | 0.036 |  |
|  |  |  | Cold-Short Day | 1.405 | 0.173 |  |
|  |  |  | Warm-Long Day | -2.377 | 0.026 |  |
|  |  |  | Warm-Short Day | -2.302 | 0.030 |  |
|  |  |  |  |  |  |  |
| Red-billed Leothrix | 20 | ΔBMR | M_b_ | -0.145 | 0.887 | Cui et al. 2019 |
| *Leothrix lutea* |  |  | BMR | 1.372 | 0.189 |  |
|  |  |  | TempGrp | -1.634 | 0.122 |  |
|  |  |  |  |  |  |  |
| Black-capped Chickadee | 24 | ΔBMR | M_b_ | 2.348 | 0.0293 | Dubois et al. 2016 |
| *Poecile atricapillus* |  |  | BMR | -1.490 | 0.152 |  |
|  |  |  | TempGrp | -0.658 | 0.518 |  |
|  |  |  |  |  |  |  |
|  | 24 | ΔM_sum_ | M_b_ | -0.088 | 0.930 |  |
|  |  |  | BMR | -0.557 | 0.584 |  |
|  |  |  | TempGrp | -0.889 | 0.385 |  |
|  |  |  |  |  |  |  |
|  | 24 | ΔScope | M_b_ | -0.858 | 0.401 |  |
|  |  |  | BMR | 0.311 | 0.759 |  |
|  |  |  | TempGrp | -0.771 | 0.450 |  |
|  |  |  |  |  |  |  |
| House Sparrow | 47 | ΔBMR | M_b_ | -1.418 | 0.163 | Swanson et al. 2020 |
| *Passer domesticus* |  |  | BMR | 2.790 | 0.008 |  |
|  |  |  | TempGrp | -0.439 | 0.663 |  |
|  |  |  |  |  |  |  |
|  | 47 | ΔM_sum_ | M_b_ | 0.177 | 0.860 |  |
|  |  |  | BMR | -0.974 | 0.336 |  |
|  |  |  | TempGrp | -0.561 | 0.578 |  |
|  |  |  |  |  |  |  |
|  | 47 | ΔScope | M_b_ | 0.253 | 0.801 |  |
|  |  |  | BMR | -0.944 | 0.350 |  |
|  |  |  | TempGrp | -0.240 | 0.811 |  |
|  |  |  |  |  |  |  |
| S. Red Bishop | 39 | ΔBMR | M_b_ | -0.872 | 0.389 | van de Ven et al. 2013 |
| *Euplectes orix* |  |  | BMR | 2.069 | 0.046 |  |
|  |  |  | TempGrp | 2.633 | 0.013 |  |
|  |  |  | Location | -0.478 | 0.636 |  |
|  |  |  |  |  |  |  |
|  | 39 | ΔM_sum_ | M_b_ | 3.319 | 0.002 |  |
|  |  |  | BMR | -0.469 | 0.642 |  |
|  |  |  | TempGrp | 2.312 | 0.027 |  |
|  |  |  | Location | -0.509 | 0.614 |  |
|  |  |  |  |  |  |  |
|  | 39 | ΔScope | M_b_ | 3.264 | 0.003 |  |
|  |  |  | BMR | -1.449 | 0.157 |  |
|  |  |  | TempGrp | 1.260 | 0.216 |  |
|  |  |  | Location | -0.843 | 0.405 |  |
|  |  |  |  |  |  |  |
| Snow Bunting | 14 | ΔBMR | M_b_ | 2.255 | 0.048 | Dubois et al. 2016 |
| *Plectrophenax nivalis* |  |  | BMR | 1.221 | 0.250 |  |
|  |  |  | TempGrp | 1.157 | 0.274 |  |
|  |  |  |  |  |  |  |
|  | 14 | ΔM_sum_ | M_b_ | 1.590 | 0.143 |  |
|  |  |  | BMR | 1.371 | 0.200 |  |
|  |  |  | TempGrp | 2.938 | 0.015 |  |
|  |  |  |  |  |  |  |
|  | 14 | ΔScope | M_b_ | 2.157 | 0.056 |  |
|  |  |  | BMR | 1.022 | 0.331 |  |
|  |  |  | TempGrp | 2.209 | 0.052 |  |
|  |  |  |  |  |  |  |
| White-throated Sparrow | 20 | ΔBMR | M_b_ | -0.523 | 0.608 | Dubois et al. 2016 |
| *Zonotrichia albicollis* |  |  | BMR | -0.067 | 0.947 |  |
|  |  |  | TempGrp | 0.724 | 0.480 |  |
|  |  |  |  |  |  |  |
|  | 20 | ΔM_sum_ | M_b_ | 1.673 | 0.114 |  |
|  |  |  | BMR | -0.806 | 0.432 |  |
|  |  |  | TempGrp | -0.221 | 0.828 |  |
|  |  |  |  |  |  |  |
|  | 20 | ΔScope | M_b_ | 1.531 | 0.145 |  |
|  |  |  | BMR | -0.720 | 0.482 |  |
|  |  |  | TempGrp | -0.288 | 0.777 |  |
|  |  |  |  |  |  |  |
| White-throated Sparrow | 28 | ΔBMR | M_b_ | -1.671 | 0.108 | Barcelo et al. 2017 |
| *Zonotrichia albicollis* |  |  | BMR | 3.824 | < 0.001 |  |
|  |  |  | TempGrp | -0.478 | 0.637 |  |
|  |  |  | DietGrp | -2.385 | 0.026 |  |
|  |  |  |  |  |  |  |
|  | 28 | ΔM_sum_ | M_b_ | 0.205 | 0.839 |  |
|  |  |  | BMR | -0.212 | 0.834 |  |
|  |  |  | TempGrp | 3.323 | 0.003 |  |
|  |  |  | DietGrp | 0.588 | 0.562 |  |
|  |  |  |  |  |  |  |
|  | 28 | ΔScope | M_b_ | 0.798 | 0.433 |  |
|  |  |  | BMR | -1.105 | 0.321 |  |
|  |  |  | TempGrp | 2.596 | 0.016 |  |
|  |  |  | DietGrp | 0.850 | 0.404 |  |
|  |  |  |  |  |  |  |
| Dark-eyed Junco | 49 | ΔBMR | M_b_ | 1.154 | 0.254 | Stager et al. 2020 |
| *Junco hyemalis* |  |  | BMR | -1.002 | 0.312 |  |
|  |  |  |  |  |  |  |
|  | 49 | ΔM_sum_ | M_b_ | 1.351 | 0.183 |  |
|  |  |  | BMR | 1.960 | 0.056 |  |
|  |  |  |  |  |  |  |
|  | 49 | ΔScope | M_b_ | 1.914 | 0.062 |  |
|  |  |  | BMR | 3.123 | 0.003 |  |
|  |  |  |  |  |  |  |
| Dark-eyed Junco | 48 | ΔBMR | M_b_ | 0.682 | 0.499 | Stager et al. 2021 |
| *Junco hyemalis* |  |  | BMR | -0.646 | 0.522 |  |
|  |  |  | *J. h. dorsalis* | 0.088 | 0.931 |  |
|  |  |  | *J. h. palliatus* | -0.392 | 0.697 |  |
|  |  |  | *J. h. shufelti* | 0.083 | 0.934 |  |
|  |  |  | *J. h. thurberi* | -0.011 | 0.991 |  |
|  |  |  |  |  |  |  |
|  | 48 | ΔM_sum_ | M_b_ | 1.376 | 0.176 |  |
|  |  |  | BMR | -0.854 | 0.398 |  |
|  |  |  | *J. h. dorsalis* | -0.177 | 0.861 |  |
|  |  |  | *J. h. palliatus* | -0.985 | 0.331 |  |
|  |  |  | *J. h. shufelti* | -1.620 | 0.113 |  |
|  |  |  | *J. h. thurberi* | -0.516 | 0.609 |  |
|  |  |  |  |  |  |  |
|  | 48 | ΔScope | M_b_ | 1.251 | 0.218 |  |
|  |  |  | BMR | -0.594 | 0.556 |  |
|  |  |  | *J. h. dorsalis* | -0.105 | 0.917 |  |
|  |  |  | *J. h. palliatus* | -0.860 | 0.395 |  |
|  |  |  | *J. h. shufelti* | -1.279 | 0.208 |  |
|  |  |  | *J. h. thurberi* | -0.440 | 0.662 |  |

**Table S2**. Results of correlation analyses of flexibility in BMR (ΔBMR) vs. flexibility in M_sum_ (ΔM_sum_) or metabolic scope (ΔScope). Pearson’s correlation coefficients (*r*) and *P*-values for correlations of ΔBMR with ΔM_sum_ (*r* Msum and *P* Msum) and ΔScope (*r* Scope and *P* Scope) are listed.

| **Species** | **n** | ***r* M_sum_** | ***P* M_sum_** | ***r* Scope** | ***P* Scope** | **Reference** |
| --- | --- | --- | --- | --- | --- | --- |
| Black-capped Chickadee | 24 | 0.104 | 0.629 | 0.143 | 0.504 | Dubois et al. 2016 |
| House Sparrow | 47 | 0.032 | 0.833 | 0.049 | 0.746 | Swanson et al. 2020 |
| Southern Red Bishop | 39 | 0.193 | 0.238 | 0.072 | 0.664 | Van de Ven et al. 2013 |
| Snow Bunting | 14 | 0.009 | 0.975 | 0.046 | 0.875 | Dubois et al. 2016 |
| White-throated Sparrow | 20 | 0.043 | 0.856 | 0.178 | 0.453 | Dubois et al. 2016 |
| White-throated Sparrow | 28 | 0.242 | 0.214 | 0.190 | 0.332 | Barceló et al. 2017 |
| Dark-eyed Junco | 49 | 0.175 | 0.230 | 0.091 | 0.534 | Stager et al. 2020 |
| Dark-eyed Junco | 48 | 0.033 | 0.825 | 0.042 | 0.777 | Stager et al. 2021 |
